# Unravelling the quality of HIV counselling and testing services in the private and public sectors in Zambia

**DOI:** 10.1093/heapol/czt036

**Published:** 2014-07-07

**Authors:** Ilana Ron Levey, Wenjuan Wang

**Affiliations:** ^1^International Health Division, Abt Associates Inc., 4550 Montgomery Avenue, Suite 800N, Bethesda, MD 20814, USA and ^2^International Health and Development Division, ICF Macro, 11785 Beltsville Drive, Calverton, Maryland, MD 20705, USA

**Keywords:** HIV counselling and testing, HIV prevention services, private sector, Zambia

## Abstract

**Background** Despite the substantial investment for providing HIV counselling and testing (VCT) services in Zambia, there has been little effort to systematically evaluate the quality of VCT services provided by various types of health providers. This study, conducted in 2009, examines VCT in the public and private sectors including private for-profit and NGO/faith-based sectors in Copperbelt and Luapula.

**Methods** The study used five primary data collection methods to gauge quality of VCT services: closed-ended client interviews with clients exiting VCT sites; open-ended client interviews; interviews with facility managers; review of service statistics; and an observation of the physical environment for VCT by site. Over 400 clients and 87 facility managers were interviewed from almost 90 facilities. Sites were randomly selected and results are generalizable at the provincial level.

**Results** The study shows concerning levels of underperformance in VCT services across the sectors. It reveals serious underperformance in counselling about key risk-reduction methods. Less than one-third of clients received counselling on reducing number of sexual partners and only approximately 5% of clients received counselling about disclosing test results to partners. In terms of client profiles, the NGO sector attracts the most educated clients and less educated Zambians seek VCT services at very low rates (7%). The private for-profit performs equally or sometimes better than other sectors even though this sector is not adequately integrated into the Zambian national response to HIV.

**Conclusion** The private for-profit sector provides VCT services on par in quality with the other sectors. Most clients did not receive counselling on partner reduction or disclosure of HIV test results to partners. In a generalized HIV epidemic where multiple concurrent sexual partners are a significant problem for transmitting the disease, risk-reduction methods and discussion should be a main focus of pre-test and post-test counselling.

**KEY MESSAGES**

- The quality of HIV counselling and testing services in the private for-profit sector is on par with other sectors and for some key variables, exceeds the quality of other sectors.
- All sectors are underperforming in the quality of key risk-reduction counselling elements including counselling on partner reduction and disclosure of results to partners.
- Clients select the type of VCT site (public, private for-profit or NGO) primarily based on geographic proximity rather than price or fear of stigma.

## Introduction

Zambia’s HIV epidemic is significant and generalized, with an adult prevalence rate of 14.3% (ZDHS 2007). Since the rate of new HIV infections in Zambia is surpassing the human and financial resources available to successfully treat all new and existing HIV cases, more effective HIV prevention interventions are necessary. Voluntary counselling and testing (VCT), an entry-point to HIV/AIDS services and an opportunity to receive risk-reduction counselling, has been heavily promoted in Zambia with the support of major international donors. For example, from 1 October 2010 to 30 September 2011, 1 877 800 individuals received HIV counselling and testing services funded by the President’s Emergency Plan for AIDS Relief (PEPFAR; PEPFAR, 2010). Despite the substantial investment for providing VCT in Zambia, there has been little effort to systematically evaluate the quality of VCT services provided by various types of health providers.

The private sector in Zambia, including for-profit, not-for-profit and faith-based actors, is an important provider of health services in Zambia, although there is less information about both the size and quality of the private sector than the public. As of 2004, the government of Zambia owns about 86% of all health facilities (Mudenda *et al.* 2008), but there is a growing number of private sector options, including 524 private for-profit facilities registered with the Medical Council of Zambia. Much of the private for-profit sector initially evolved to serve mining company workers in the Copperbelt and in urban areas with higher levels of formal employment. Today, a range of private for-profit providers from large-scale mining hospitals to small-scale private providers exist and play an important role in providing HIV and other health care services to Zambians despite the fact that they have fewer training opportunities and little representation in formulating health policy (Mudenda *et al.* 2008). Given the increasing demands for HIV prevention and resources needed for HIV treatment in many high prevalence countries, the private for-profit sector may be increasingly called upon to contribute to Zambia’s national AIDS response (Wang *et al.* 2011). In addition, some patients may perceive private for-profit facilities as more confidential and more sensitive to patient needs (Sargent *et al.* 2009). Thus, it is important to understand both the role and the quality of VCT services in the private sector in order to further incorporate private providers into national HIV prevention efforts.

In 2012, the Government of Zambia recommended universal testing for HIV (Zambia Online 2012) and explicitly recommended that both public and private channels would need to contribute to such a broad initiative. Although other types of counselling and testing initiatives, such as provider-initiated counselling and testing, are increasingly important to reach universal testing, VCT was the original mechanism for HIV testing deployed in Zambia and much of Africa (WHO 2007). Examining the quality of VCT services may reveal barriers and gaps in other types of counselling and testing mechanisms that slow progress towards effective universal testing.

The literature review revealed that there were no previous efforts to evaluate the quality of VCT provided by the private sector over other types of facilities. Previous quality assessments focused on one sector, franchise or NGO provider (ZPCT 2008) and often used client satisfaction as the measurement of quality of VCT services. However, clients, particularly in the developing world, tend to report overall high levels of satisfaction with health services (Sitzia and Wood 1997), even if those services are of low quality. Finally, some researchers have shown that the interpersonal aspects of care are often regarded by clients as the principal component of satisfaction (Blanchard *et al.* 1990). Since the efficacy of VCT as an intervention relies heavily on the quality of risk-reduction counselling offered in conjunction with an HIV test, the interpersonal attributes of a counsellor (e.g. warmth or lack of criticism about sexual behaviour) may be valued more heavily by clients, particularly women, than the content of the counselling they receive.

Over the last 10 years, there have been numerous efforts to set international guidelines and standards for the provision of high-quality VCT, mainly by the World Health Organization (WHO) and the United Nations Joint Programme on HIV/AIDS (UNAIDS). In March 2006, the Zambian Ministry of Health (MOH) adapted these international guidelines and developed the Zambian National Guidelines for HIV Counselling and Testing. All health facilities, including from the private for-profit, NGO and faith-based sectors, are expected to adhere to these standards which emphasize patient confidentiality, risk assessment for HIV transmission, disclosure of HIV test results on the same day as testing, referrals for follow-on care services, and satisfactory physical or structural environments to perform VCT safely.

To address the information gap concerning both the private sector’s role and the overall quality of VCT services in Zambia, this study examines VCT services across the public, private for-profit and NGO/faith-based sectors in two provinces in Zambia, Copperbelt and Luapula.

## Methods

### 

 

#### Study area

We selected Copperbelt and Luapula as our study provinces as they are United Agency for International Development (USAID)-supported provinces and have adequate number of private for-profit facilities and include both an urban (Copperbelt) and a rural (Luapula) area. This selection provides a useful opportunity for rural and urban comparison. HIV prevalence among adults is approximately 17% in Copperbelt and 13% in Luapula (ZDHS 2007) and corresponds with the overall national trend of higher HIV prevalence in urban areas (20%) as compared to rural (10%). This study was conducted by Abt Associates in 2009 under the USAID-funded Private Sector Partnerships-*One* project as part of a 5 year effort to both assess and improve the provision of reproductive health and HIV/AIDS services in the private for-profit sector in the developing world.

#### Ethical clearance

All participants provided informed consent and this research protocol was approved by both the Abt Associates’ Institutional Review Board (IRB) and ERES Converge, a Zambian private IRB approved by the MOH. Written informed consent was obtained for all participants.

### Sampling

#### Sampling of facilities

The target population for this survey is all health facilities that provide VCT services in the two selected provinces. Some health facilities in the two provinces are stand-alone VCT providers, although the majority of health facilities offer VCT along with a wide variety of other health services. Both types of facilities are included in the target facility population. Additionally, only static VCT sites were eligible for inclusion in this study, since mobile VCT is not widely utilized across the health system.

In each province, we compiled a full list of all health providers and facilities offering VCT services from a variety of sources. There is not a comprehensive or accurate master list of all registered VCT sites in Zambia, particularly for the private for-profit sector. Key sources checked during the compilation of the sample include the Zambia Voluntary Counselling and Testing Services; the Medical Council of Zambia; the USAID-funded Zambia Prevention, Care and Treatment Partnership project; the Comprehensive HIV/AIDS Management Programme; the Society for Family Health; and the Christian Health Association of Zambia.

Based on these various sources, we compiled a sampling frame of 373 health facilities providing VCT services in Copperbelt and 157 facilities in Luapula, for a total of 530 facilities. A separate list was compiled for each of the four sectors: public, private for-profit, NGO and faith-based facility. For each type of sector, equal probability systematic sampling was used to select the facility samples.

We originally sampled 28 facilities from the public sector. The list of public facilities was first sorted by type of facility, including hospitals and health centres for each province. The facilities were then sorted by estimated size (large, medium and small). Facilities of unknown size were considered as a separate category. Large hospitals were purposively selected so that we could ensure the maximum number of clients possible in our study. After we sorted the public sector list, equal probability systematic sampling was used with a fractional sampling interval to ensure that we selected the exact number of facilities that we required. We sampled facilities from the private for-profit, NGO and faith-based sectors using a similar methodology.

The final list of facilities visited included a smaller number of private for-profit facilities than anticipated. In the course of data collection we discovered that *no* private for-profit facilities in Luapula actually provided VCT services, even though nine were registered as such.

#### Sampling of exiting clients

Clients over the age of 18 who had received HIV counselling, testing and HIV test results at a particular facility on the same day were approached for exit interviews during a 6-h data collection period at each selected facility. Among 65 of 87 facilities sampled, we successfully recruited clients for exit interviews. Due to the very low VCT client volume in the rest of 22 facilities on the day of data collection, we failed to recruit any clients.

Table 1 shows the universe of facilities identified by sector; the number of facilities selected by province; and the number of clients receiving VCT that were interviewed during data collection. The final sample included 87 health facilities and 364 clients.

**Table 1:** Distribution of the sample of sites by province and sector

	Copperbelt			Luapula		
	Universe of sites	No. of sampled sites with VCT clients interviewed	No. of clients interviewed	Universe of sites	No. of sampled sites with VCT clients interviewed	No. of clients interviewed
Public	121	17	71	119	11	66
Private	166	13	84	9	0	0
NGO	24	9	79	13	5	20
Faith based	14	3	8	10	2	18
Unknown	50	5	18	7	0	0
TOTAL	373	47	260	157	18	104

#### Interview

Three types of interview instruments were used in the study: a closed-ended client exit interview, an open-ended client exit interview and a facility assessment instrument. In each selected facility, Facility Managers or Clinic Owners were first approached and interviewed to capture information related to the VCT services offered at each facility and measure quality assurance procedures. The facility assessment instrument also contained a review of the last month’s VCT registry and service statistics, and a brief observation of the physical environment of the VCT site. The client exit interviews aimed to understand the VCT experience from the perspective of the client, as well as their demographics and socio-economic profiles. The survey instrument for the client exit interview was designed according to the Zambian National Guidelines and relates to structural and process elements of VCT quality, with a particular emphasis on counselling, future behaviour change intention, and referral uptake intention. The survey instrument asked clients both about the pre-test and post-test counselling that they received during their visit. Per Zambian National Guidelines, all clients receiving VCT should undergo pre-test counselling, testing, confirmation of test results and post-test counselling during one visit. However, the instruments did not ask clients to report the results of their HIV test. The instruments were translated into Bemba.

#### Data analysis

Data analysis was conducted with commercially available *STATA 10* statistical software. Separate analysis was conducted for client and facility data. Sample weights were computed according to the probability of selection. All results were adjusted accordingly. The analysis is primarily descriptive. Design-based Pearson’s chi-square tests are used to test the significance of differences across groups. Due to the similar nature of NGO and faith-based sectors, we combine together these two groups for comparisons.

The open-ended client interviews were transcribed and imported into commercially available NVIVO 7, a software package designed to facilitate rigorous qualitative data analysis. We coded the interview transcripts by emerging themes and sorted the data by sector and province. The open-ended instrument did not include extensive prompts; clients were encouraged to respond in their own words about their experience with VCT services.

## Results

### 

 

#### Socio-demographic profile of clients in each sector

Table 2 shows the demographic characteristics of interviewed clients. Results show that women are more likely than men to use public sector facilities vs private for-profit and NGO/faith-based facilities for VCT. The oldest clients are most likely to visit private for-profit sites, whereas the younger clients tend to visit NGO sites. There are also significant differences in the education levels of clients, with the higher educated clients more likely to use NGO and private for-profit facilities. More importantly, across all sectors, clients with no education or only primary levels of education are less likely to access VCT services: on average, only 6% of clients receiving VCT had no or primary levels of education. There is no statistically significant difference in utilization of a particular sector for VCT according to self-rated socio-economic status.

**Table 2:** Client’s socio-demographic characteristics and transportation profiles

	Public (%)	Private (%)	NGO/Faith based (%)
Gender*			
Male	33.3	42.9	48.9
Female	66.7	57.1	51.1
Median age (years)	31.0	38.0	28.0
Age groups***			
15–24	27.6	14.2	29.2
25–34	36.8	25.0	31.4
35–44	28.6	39.1	24.6
45+	7.0	21.6	14.8
Education levels***			
None	2.6	0.0	0.9
Primary	3.8	2.4	3.8
Secondary	48.9	29.8	40.9
Higher than			
secondary	37.6	45.4	46.2
Do not know	7.0	22.4	8.3
Self-rated socio-economic status			
Lower	30.3	31.2	43.9
Middle	67.6	61.8	49.1
Upper	2.1	7.1	6.7
Mode of travel to the VCT site***			
Walked	59.4	65.6	66.6
Took public transportation	11.7	20.2	13.3
Used personal car	2.1	9.4	2.3
Taxi	3.2	2.4	3.6
Bicycle	21.0	1.2	11.9
Other	2.6	1.2	2.3
Time taken to travel to site**			
Less than 30 min	43.5	63.0	53.8
30 min	40.0	33.4	22.9
1–2 h	9.7	1.2	9.9
More than 2 h	7.0	2.4	13.4
Paid for transportation today			
Yes	18.7	30.9	23.7
No	81.3	69.1	76.3
Total number of clients	137	84	125

***P < 0.001; **P < 0.05; *P < 0.1

We also gathered information on mode of travel, expense and time spent travelling to the VCT site to ascertain how geographically proximate the VCT services were for clients. The results show that overall most clients walked to their VCT facility and spent less than 30 min commuting. However, there are significant differences by sector in terms of geographic proximity: public transportation was used most often in the private sector. Private clients had a significantly shorter commuting time but were more likely to pay for transportation than public or NGO/faith-based clients.

We asked clients to report how much they paid out-of-pocket (OOP) to receive their VCT service. More NGO clients paid OOP for service than private sector clients, but the median amount they paid ($.21) was much lower than that paid by private sector clients ($7.50). As expected, very few clients paid OOP for VCT services in the public sector.

The open-ended client interviews supported that proximity or convenience is an essential factor for considering a facility for VCT and outweighed concerns about price.

#### Contents of counselling

**Figure 1:**
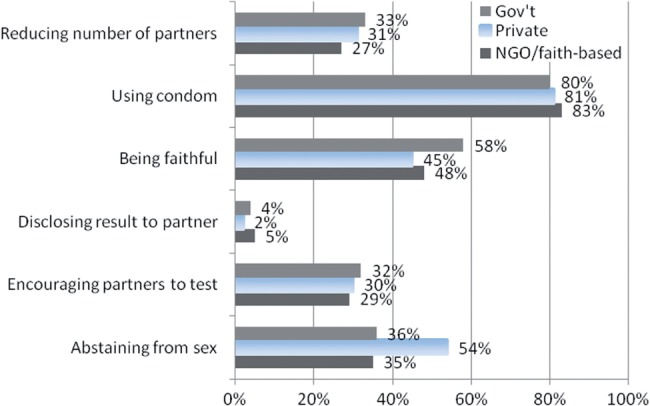
Risk-reduction methods discussed with counsellor for reducing exposure to HIV.

The survey asked about contents of counselling at both the pre-HIV test and the post-HIV test stage, as specified by the National Guidelines. Figure 1 shows the percentage of clients in each sector who reported specific HIV risk-reduction methods discussed with the counsellor during pre- or post-HIV test. It demonstrates underperformance in counselling on pertinent key risk-reduction methods across all sectors. On average, less than one-third of clients across the sectors received counselling on reducing their number of sexual partners. Only approximately 5% of clients or fewer received counselling about disclosing their test results to their partners. Using condoms, being faithful and abstaining from sex are relatively more covered compared to other risk-reduction methods. The private sector performs similarly to the other sectors in counselling on the majority of risk-reduction methods, although it outperformed the other two types of sectors in counselling on abstaining from sex.

We asked clients questions about their VCT service in terms of clarity of the HIV test result explanation and if the counsellor answered client questions with enough detail. The results show that 85% of clients in the private sector reported ‘very clear’ compared to 69% and 70% in the public and NGO/faith-based sectors, respectively.

Open-ended interviews asked clients to describe, unprompted, new information they had learned during their pre- and post-test counselling sessions. Clients reported a range of counselling topics and information that do not adhere to the Zambian National Guidelines for VCT and are not germane to the generalized Zambian HIV epidemic driven largely by heterosexual transmission.

For instance, clients across all sectors and both provinces reported that they were counselled on preventing blood transmission of HIV via razor blades or blood transfusion. Forty-five percent of public sector clients in both Copperbelt and Luapula reported, unprompted, an emphasis on blood transmission of HIV during their pre-test counselling sessions. In fact, blood transmission was the most frequently discussed topic during pre-test counselling across all sectors. Additionally, 15% of clients reported receiving pre-test counselling about living ‘positively’ as an HIV-positive individual. For instance, a public sector client reported being told ‘that I can live a long life as long as I get tested and start taking the right medication and right food.’ Another public sector client believed ‘I can actually live longer than the person who is negative.’ The National Guidelines, however, state that pre-test counselling should focus more on risk-reduction methods and an assessment of sexual behaviour than on strategies for responding to an HIV-positive test result.

#### Waiting times for VCT

Waiting times are an important component of overall VCT quality. Long waiting times, particularly while waiting for an HIV test result, may deter clients from learning their HIV status. There is a significant difference between sectors in the initial wait to see a counsellor for pre-testing counselling. The NGO sector recorded the shortest average wait time—29 min—whereas clients in the private sector waited the longest—55 min. There were no significant differences between sectors in the median amount of time spent by clients in their pre-test or post-test counselling sessions, or in waiting for an HIV test result. There were relatively low wait times for HIV test results after testing, and post-test counselling sessions were substantially shorter than pre-test counselling sessions.

#### Future intention for behaviour change

Since this study was not longitudinal, we could not determine whether or not clients changed their sexual behaviour or reduced risk for HIV transmission following their VCT services. However, we did measure the *intention* of clients to change their behaviour and/or retest for HIV following their VCT session.

Table 3 shows few significant differences across sectors for intention to change behaviour. Positively, most clients across all sectors self-reported an intention to test for HIV again in the next 12 months. Relatively few clients intended to reduce their number of sexual partners in the future; this number was only 9.6% for private for-profit sector clients despite that about one-third of clients in this sector reported counselling on reducing number of partners. This low level of intention to reduce the number of sexual partners in the future may be linked to current low levels of self-reported multiple sexual partners and a perceived disinclination to acknowledge multiple sexual partners, both in the future or the present. More than half of clients intended to use condoms in the future. It is not surprising that fewer than 5% of clients intended to disclose their HIV test results to potential partners, given the lack of counselling across the sectors relating to this key risk-reduction method. Although there is evidence of self-report bias in the literature in terms of intention to change behaviour (Brener *et al.* 2003), we see wide variance in levels of self-reported intention to change. This variance suggests that clients do consider and weigh each behaviour type separately and do not have high levels of intention to change sexual and HIV risk prevention behaviour across the board. The contradictions in level of behaviour change intention—particularly around the lower levels of intention to reduce sexual partners as compared to intention to remain faithful—may reduce client risk perceptions and impede efforts towards universal testing.

**Table 3:** Behaviour change intention in the future

Future test intention	Public (%)	Private (%)	NGO/faith based (%)
Intention to test for HIV again in the next 12 months	92.6	88.2	94.7
Likely to follow-up at referred facility	93.1	96.3	91.2
Future behaviour change			
Reduce number of partners**	25.0	9.6	22.4
Use condoms	56.7	57.1	67.7
Be faithful*	58.0	44.0	49.9
Disclose results	1.8	2.4	4.9
Encourage others to test	30.9	32.0	26.2
Total number of clients	137	84	125

***P < 0.001; **P < 0.05; *P < 0.1

### Facility-level results

This section presents findings based on interviews with Facility Managers; reviews of VCT service statistics at each facility; and observations of the VCT physical environment at each facility.

Table 4 shows differences in client load rates across the sectors. There are no significant differences between sectors in the mean number of clients visiting each facility for VCT services in the past year. However, NGO/faith-based facilities have by far the highest percentage of clients visiting for VCT services, which suggests that NGO facilities offer the most specialized HIV services as opposed to general medical services. Public facilities in our sample had the lowest mean number of clients visiting specifically for VCT.

**Table 4:** Key facility statistics by sector

Client load	Public	Private	NGO/faith based
Mean number of clients per month coming for HIV VCT	53	65	69
Mean no. of outpatients per month coming for all health services	603	829	267
Number of clients post-test counselled AND received results^a^	58	120	100
Number of all clients testing HIV positive^a^	18	21	21
Total number of facilities	38	28	21

^a^During the month under review (March 2009)

The review of key HIV service statistics for March 2009 at each facility reveals that in the private for-profit sector, 120 clients received HIV post-test counselling and a test result, whereas in the public sector only 58 clients did so. This finding suggests that the private sector is well utilized for VCT services by clients, who tend to stay to receive their HIV test result. The private sector also had 21% of clients coming for VCT services testing HIV positive. This finding is noteworthy since the private for-profit is the sector least utilized or funded by international donors or the Zambian government for HIV prevention efforts although it appears to have high utilization for HIV prevention services.

## Discussion

Using mixed quantitative and qualitative methods of data collection, this study examines the VCT services provided by public, private for-profit and NGO/ faith-based sectors in two provinces in Zambia. This study is the first effort to systematically assess and compare VCT services across multiple sectors in Zambia.

The study is subject to several limitations. Firstly, data from this study are valid and generalizable only at the provincial level, for Copperbelt and Luapula. We cannot generalize these results to other areas of Zambia. Secondly, clients were recruited for exit interviews during a 6-h time window in the sampled facility. People interviewed may not be representative of the population in the province. Also clients choosing to access VCT are self-selecting and may have health behaviours different from the rest of the population. Additionally, we failed to recruit VCT clients in some facilities sampled due to the extremely low client volume on the day of the data collection. Thirdly, the sampling frame of VCT facilities was constructed based on a number of sources. There is a possibility that some facilities, particularly from the private for-profit sector, are omitted due to not being registered with any of the sources we had. Fourthly, we chose to measure quality through client recollections of their immediate counselling and testing session, a review of service statistics, and physical environment observations, and interviews with facility managers. Due to concerns about bias, confidentiality and protection of HIV test results, we did not directly observe VCT counsellors interact with clients.

Although our analysis was inevitably constrained by these limitations, some noteworthy findings have emerged that have important policy and program implications. We find that there are important demographic differences between the clients choosing to access VCT services in each sector. These findings suggest that HIV prevention services in each sector could be targeted to particular segments of the population. Note that, across the sectors, very few clients with no or primary levels of education sought out VCT services. However, HIV prevalence increases with education for both women and men in Zambia (ZDHS 2007) so less-educated clients may have lower HIV risk perceptions.

The fact that clients emphasize proximity in seeking a facility for VCT service indicates that clients do not fear being recognized at VCT sessions, either by providers or by other community members. The preference for VCT services ‘close to home’ suggests that proximity of the VCT site is an important factor in choosing a facility. This finding is noteworthy since many studies across sub-Saharan Africa (Banteyerga *et al.* 2003) suggest that clients prefer to access HIV services away from their local communities due to concerns of being recognized and fear of resulting stigma or discrimination. In the examination of process, structural and outcome dimensions of VCT services, we find concerning levels of underperformance in VCT across the entire health system. Both qualitative and quantitative data from all sectors revealed serious underperformance in counselling about key risk-reduction methods, including sexual partner reduction and disclosure of HIV test results to partners. While most counsellors are addressing condom usage during pre- and post-test counselling sessions, there is little evidence of a widespread effort to address either the situation of multiple, concurrent sexual partnerships or the necessity to disclose HIV test results to sexual partners.

Many clients reported receiving inappropriate counselling messages regarding HIV transmission that emphasize needle exchange, blood contact or tattoos. This inclusion of counselling content inappropriate for the Zambian context indicates that training materials may not be sufficiently tailored to the epidemiological reality of the Zambian epidemic. In addition, discussions around concurrency and multiple sexual partners may be particularly difficult for counsellors given low levels of client willingness to disclose about these partners. In November 2009, Rupiah Banda, President of Zambia, publicly stated that ‘multiple, concurrent partnerships are the leading cause of HIV infection in Zambia (PlusNews 2009).’ Thus, the content of VCT sessions in Zambia should better address the risks of concurrency.

In many of the quality variables we examined, no clear patterns existed among the sectors; i.e. no one sector emerges as providing overwhelmingly higher quality services than another. The private for-profit sector, however, did perform on par with the other sectors in VCT quality. This performance is noteworthy given the lack of inclusion of the private for-profit sector in most HIV prevention training opportunities in Zambia. Through our study, we know that private providers do provide VCT at comparable rates, often experience high client load and more clients received post-test counselling and HIV test results in the private for-profit sector than in any other sector. These findings suggest that the Zambian government should look to maximize the contributions of the private for-profit sector and should view this sector as an important resource for the country in meeting the pressing needs of HIV prevention.

## Conclusion

These findings help to dispel certain myths and misconceptions regarding VCT services in Zambia. No one sector emerges as providing overwhelmingly higher quality services than another; and, overall, rural sites perform on par in quality with the urban sites. However, the findings reveal less than optimal counselling practices across the sectors. The study findings can be used by the MOH in Zambia to improve training curricula for VCT counsellors and ensure ongoing supportive supervision of VCT practices. In addition, this study contributes to the evidence base by documenting and quantifying for the first time both the extent of VCT provision by the private for-profit sector and the quality of that provision. By further incorporating this important sector into training, planning and operationalizing HIV strategies, Zambia will be able to offer high-quality VCT services to a greater number of Zambians. Finally, the study sheds light on some important patterns and barriers towards higher uptake of VCT in Zambia. Understanding these barriers is a key first step towards an effective path to universal HIV testing in Zambia.

## Ethical approval

The study received ethical approval from the Abt Associates Institutional Review Board (IRB; IRB review #0413) as well as the Zambian ERES Converge IRB (IRB approval #2009-Feb-001).

## References

[czt036-B2] Banteyerga H, Aklilu K, Laura N, Kerry M, Rohini P (2003). Exploring HIV AND AIDS Stigma and Related Discrimination in Ethiopia: Causes, Manifestations, Consequences and Coping Mechanisms.

[czt036-B4] Blanchard CG, Labrecque MS, Ruckdeschel JC, Blanchard EB (1990). Physician behaviors, patient perceptions, and patient characteristics as predictors of satisfaction of hospitalized adult cancer patients. Cancer.

[czt036-B5] Brener ND, Billy JOG, Grady WR (2003). Assessment of factors affecting the validity of self-reported health-risk behavior among adolescents: evidence from the scientific literature. Journal of Adolescent Health.

[czt036-B9] http://www.psi.org/research/smr/707-togo_mystery_client.pdf.

[czt036-B11] Mudenda D, Christopher M, Chita B, Chompolola A, Wake W (2008). Provider Purchasing and Contracting for Health Services: The Case of Zambia.

[czt036-B12] PlusNews (2009). Zambia: new infections on the rise. http://plusnews.org/report.aspx?ReportId=86946.

[czt036-B14] Sargent J, Johnson J, Majorowski M, Friedman N, Blazer C (2009). Private Sector Involvement in HIV Service Provision.

[czt036-B16] Sitzia J, Wood N (1997). Patient satisfaction: a review of issues and concepts. Social Science and Medicine.

[czt036-B17] The President’s Emergency Plan for AIDS Relief (2010). Zambia Country Page. http://www.pepfar.gov/countries/zambia/index.htm.

[czt036-B18] Wang W, Sulzbach S, De S (2011). Utilization of HIV related services from the private health sector: a multi-country analysis. Social Science & Medicine.

[czt036-B22] World Health Organization (2007). Guidance on Provider-Initiated HIV Testing and Counselling in Health Facilities.

[czt036-B24] Zambia Demographic and Health Survey (2007). Central Statistical Office (CSO), Ministry of Health (MOH), Tropical Diseases Research Centre (TDRC), University of Zambia, Macro International Inc. 2009.

[czt036-B25] Zambia Ministry of Health (2006). Zambia National Guidelines for HIV Counseling and Testing.

[czt036-B26] Zambia Online (2012). State for Universal HIV Testing. http://zambia.co.zm/news/headlines/2012/12/21/state-for-universal-hiv-testing/.

